# P18 Uncovering the role of secondary messenger signalling in *Acinetobacter baumannii* virulence

**DOI:** 10.1093/jacamr/dlae136.022

**Published:** 2024-08-23

**Authors:** Lyuboslava G Harkova, Rubén de Dios, Ronan R McCarthy

**Affiliations:** College of Health and Life Sciences, Department Of Life Sciences, Brunel University London, Uxbridge, UB8 3PH, UK; College of Health and Life Sciences, Department Of Life Sciences, Brunel University London, Uxbridge, UB8 3PH, UK; College of Health and Life Sciences, Department Of Life Sciences, Brunel University London, Uxbridge, UB8 3PH, UK

## Abstract

**Backgrounds:**

Secondary messenger signalling systems play a crucial role in bacterial capability to respond to environmental changes by mediating alternations in transcription, translation and enzyme activity. Secondary messengers are central regulators of various aspects of bacterial life including metabolism, pathogenicity, sessility and cell morphology. Indeed, these molecules are widely recognized mediators of virulence and antibiotic tolerance in well studied pathogens such as *Pseudomonas aeruginosa*, where over 40 enzymes contain domains with the predicted potential to influence secondary messenger levels. The MDR nosocomial pathogen *Acinetobacter baumannii* has become increasingly prevalent over the last 20 years with carbapenem-resistant strains surpassing *P. aeruginosa* to become the WHO top priority pathogen. Despite this, relatively little is known about the role of secondary messengers in *A. baumannii* pathogenicity.

**Methods:**

In this work, we used high-throughput screening of the *A. baumannii* AB5075 transposon mutant library to identify novel regulators of biofilm formation. Amongst the hits were genes predicted to influence secondary messenger levels. We further characterized the candidate with the strongest biofilm phenotype and uncovered a range of other phenotypes such as motility, exopolysaccharide production, virulence, antibiotic resistance and link with other signalling systems.

**Results:**

RNA-Seq analysis of a clean deletion mutant compared with a complemented strain showed that operons and genes linked to the observed phenotypes such as *pgaABCD*, *csuA/BABCDE* and type IV pili genes were differentially expressed. Moreover, antibiotic resistance and virulence in an *in vivo* model were directly affected too.

**Conclusions:**

Overall, we demonstrate the important role of secondary messenger signalling in *A. baumannii* and confirm that it modulates key phenotypes linked to virulence and antimicrobials resistance.

